# Time perspective status and associated factors among young and middle-aged women with gynecologic malignancies who have parenting responsibilities

**DOI:** 10.1016/j.apjon.2026.100994

**Published:** 2026-06-13

**Authors:** Guiyuan Ma, Cai Deng, Qiming Ding, Can Gu, Juan Peng

**Affiliations:** a Xiangya School of Nursing, Central South University, Changsha, China; b Clinical Nursing Teaching and Research Section, The Second Xiangya Hospital, Central South University, Changsha, China; c Emergency Department, The Second Xiangya Hospital, Central South University, Changsha, China

**Keywords:** Time perspective, Gynecologic malignancies, Young and middle-aged women, Sense of coherence, Social support, Psychological adaptation

## Abstract

**Objective:**

Gynecologic malignancies in young and middle-aged women severely disrupt life plans and future expectations. While Time Perspective (TP) theory offers a valuable framework for understanding psychological adaptation, research on TP status and its associated factors in this population remains scarce. This study aimed to characterize TP status and identify associated factors.

**Methods:**

A cross-sectional study was conducted among 266 young and middle-aged patients with gynecologic malignancies who were married or cohabiting, had at least one minor child, and were hospitalized across three provincial hospitals in Changsha, China. The Zimbardo Time Perspective Inventory was used to assess five TP dimensions. Guided by the Social Ecological Model, psychosocial and clinical variables were collected, and multivariable regression analyses were performed to identify factors associated with each TP dimension.

**Results:**

Participants showed relatively higher scores for Past-Positive (3.65 ± 0.53) and Future (3.62 ± 0.65) orientations, relatively lower scores for Past-Negative (2.85 ± 0.63) and Present-Hedonistic (2.89 ± 0.78) orientations, and a moderate score for Present–Fatalistic orientation (3.13 ± 0.79). Multivariable regression analyses showed that the factors associated with TP varied across dimensions. Past-Negative orientation was associated with residence, education level, employment status, sense of coherence (SOC), distress disclosure (DD), perceived social support scale (PSSS), perceived partner responsiveness (PPRS), and parenting concerns questionnaire (PCQ). Past-Positive orientation was associated with residence and cancer stage. Present-Hedonistic orientation was associated with residence, education level, occupation, employment status, medical insurance, cancer diagnosis number of children, SOC, and DD. Present–Fatalistic orientation was associated with residence, occupation, SOC, DD, PSSS, PPRS, and financial toxicity. Future orientation was associated with residence, occupation, cancer stage, SOC, and PSSS.

**Conclusions:**

This specific cohort of young and middle-aged women with gynecologic malignancies displayed a distinct temporal profile shaped by psychosocial, occupational, and residential factors. These findings suggest that internal psychological resources and relational support were associated with more adaptive future-oriented thinking, whereas structural and occupational vulnerabilities may be associated with stronger fatalistic orientation. These findings highlight the necessity of incorporating TP assessment into psychosocial screening and suggest that interventions targeting temporal reorientation may promote psychological adjustment in this population.

## Introduction

According to the International Agency for Research on Cancer (IARC) Global Cancer Observatory, approximately 1.47 million new gynecologic malignancy cases and 680,372 deaths occurred worldwide in 2022.^1^ These cancers, primarily ovarian, cervical, endometrial, and vulvar malignancies,^2^ account for 15% of all female malignancies.^1^ Diagnosed during a critical life stage involving child-rearing and career development,^1^^,^^3^ these patients face profound disruptions to fertility, female identity, and social roles.^4^, ^5^, ^6^ Despite a high prevalence of psychological distress^5^^,^^6^ in this population, significant heterogeneity in adaptation persists among those with comparable clinical profiles.^7^^,^^8^ This phenomenon suggests that internal cognitive frameworks, particularly Time Perspective (TP), may help explain these individual differences.^9^^,^^10^

TP modulates how individuals partition their temporal experiences, ultimately determining their unique adaptation trajectories.^9^^,^^10^ A cancer diagnosis often constitutes a “temporal crisis” that fractures life continuity,^11^ compelling patients to integrate an uncertain future and a traumatic past with a functionally impaired present.^9^^,^^12^^,^^13^ Therefore, the manner in which individuals cognitively organize these temporal frames may be linked to psychological resilience.^9^^,^^11^ Prior oncological studies have indicated that distinct patterns of temporal focus, such as rumination on negative past experiences or a sense of present helplessness, are closely associated with a diminished quality of life.^12^

Drawing upon Zimbardo's Time Perspective theory, this study aims to comprehensively evaluate the temporal cognitive status of this population.^10^^,^^14^ TP theory posits that an individual's orientation across five temporal dimensions (Past-Negative, Past-Positive, Present-Hedonistic, Present–Fatalistic, and Future) shapes their emotional adjustment to stressful life events.^10^^,^^14^^,^^15^ Although this framework has been applied to various cancer cohorts, revealing an imbalanced TP status,^12^^,^^13^ such as a foreshortened future horizon,^9^^,^^11^^,^^12^ further research is necessitated by the unique clinical and developmental challenges inherent to young and middle-aged women with gynecologic malignancies.^1^ Unlike other cohorts, these patients face a profound “temporal squeeze”; they must navigate biological threats to fertility and hormonal stability, while simultaneously managing peak responsibilities in career and caregiving.^10^^,^^15^^,^^16^ This diagnosis triggers a crisis that fractures multifaceted social roles in a manner distinct from older populations or non-reproductive cancers.^11^^,^^17^ Exploring TP in this specific group is, therefore, essential to understanding how these intersecting stressors reshape internal cognitive frameworks.

**Fig. 1:**
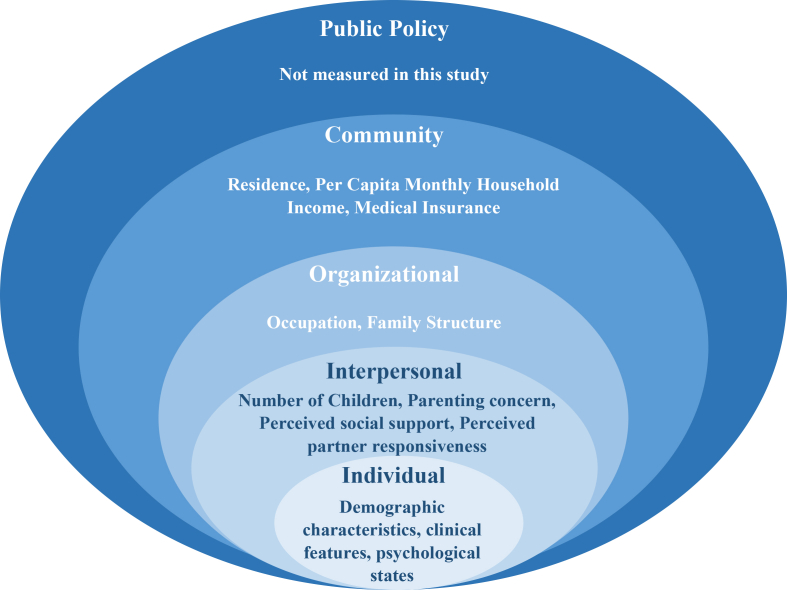
Bronfenbrenner's Social Ecological Model for identifying measurement indicators (This study did not measure policy-level factors, so this level was not included in the analysis).

To address these gaps, this study utilized Bronfenbrenner's Social Ecological Model^18^ as a holistic analytical lens (Fig. 1), emphasizing that cognitive patterns are shaped by interconnected environmental systems.^18^ Accordingly, we systematically investigated key variables across four levels: individual (e.g., sense of coherence,^19^ clinical features), interpersonal (e.g., perceived social support and partner responsiveness^20^), organizational (e.g., occupation), and community (e.g., socioeconomic context) levels. This multi-level approach allows for a comprehensive understanding of how personal resources, social interactions, and structural factors collectively shape the TP of this vulnerable cohort.

Specifically, this study focuses on married or cohabiting women with children. This recruitment strategy is based on the premise that these women sit at a unique intersection of personal recovery and intensive caregiving.^2^^,^^5^^,^^6^ This subpopulation faces distinct temporal conflicts and psychological pressures that differ significantly from those of single or childless women, thereby warranting a targeted investigation.^4^^,^^7^ Consequently, the objectives of this study were: (1) to characterize the five TP dimensions among young and middle-aged patients with gynecologic malignancies; and (2) to explore the multi-level factors, guided by the Social Ecological Model, that are associated with their TP status.

## Methods

### Participants

This cross-sectional study was conducted between August and December 2024 at the gynecologic oncology wards of three provincial hospitals in Changsha, Hunan Province, China. Participants were recruited during hospitalization via convenience sampling, and written informed consent was obtained from all participants.

Inclusion criteria comprised: (1) pathologically confirmed gynecologic malignancy (e.g., cervical, ovarian, or endometrial cancer); (2) age between 18 and 59 years;^21^ (3) being in a stable marital or cohabiting relationship (defined as a long-term, marriage-like living arrangement without legal marriage);^22^ (4) parenting at least one minor child (aged < 18 years); (5) fluency in Mandarin Chinese; and (6) full awareness of their cancer diagnosis to ensure ethical psychological assessment. Exclusion criteria included cognitive impairment precluding independent survey completion or a history of severe psychiatric disorders.

### Sample size

Based on the principle that the sample size for multivariable analysis should be 5 to 10 times the number of independent variables,^23^ and considering the 19 independent variables examined in this study, we targeted 10–15 participants per variable. This yielded a theoretical requirement of 190–285 cases. Accounting for a 20% anticipated invalid response rate, the final required sample size was calculated to be 228–342 participants.

### Measurements

#### Demographic and clinical characteristics

A self-designed structured questionnaire was used to collect data on age, number of children, age of the youngest child, residence, education level, occupation, employment status, medical insurance, family structure, cancer diagnosis, cancer stage, treatment phase, and per capita monthly household income (¥) (Regarding the treatment phase, patients were classified into active, stable, or palliative phases based on their current clinical management status, serving as an indicator of clinical status rather than overall disease duration.)

#### Time perspective (TP)

The 25-item short-form Chinese version of the Zimbardo Time Perspective Inventory (ZTPI)^14^ was used to evaluate five dimensions: Past-Negative, Past-Positive, Present-Hedonistic, Present–Fatalistic, and Future.^14^ Past-Negative reflects a negative, aversive attitude toward the past, often associated with trauma or regret (7 items: 8, 12, 13, 15, 16, 23, 25). Past-Positive represents a warm, nostalgic attitude toward the past (6 items: 1, 2, 5, 7, 9, 14). Present-Hedonistic indicates a focus on immediate pleasure and excitement, with a reluctance to sacrifice present enjoyment for future gains (4 items: 3, 11, 21, 22). Present–Fatalistic pertains to a helpless and hopeless attitude toward the future and life, characterized by the belief that life is controlled by uncontrollable forces (3 items: 17, 18, 19). Future measures a focus on planning for and achieving future goals, characterized by the ability to delay gratification (5 items: 4, 6, 10, 20, 24). All items are rated on a five-point Likert scale, with higher scores indicating stronger endorsement of the corresponding TP dimension. In this study, Cronbach's alpha coefficients for the five subscales ranged from 0.656 to 0.783.

#### Parenting concerns Questionnaire (PCQ)

The Parenting Concerns Questionnaire (PCQ)^24^ assessed cancer-related concerns across three domains: emotional impact on the child, practical impact on the child, and co-parenting concerns. Items are rated on a five-point Likert scale (1 = not at all to 5 = very much), with higher mean scores indicating greater parenting concern. In the present study, the Cronbach's alpha coefficient for the total scale was 0.912.

#### Sense of coherence (SOC)

The 13-item SOC Scale (SOC-13)^25^^,^^26^ assessed participants' global orientation toward life as comprehensible, manageable, and meaningful. Items are rated on a seven-point Likert scale (1 = very often to 7 = never), with total scores ranging from 13 to 91; higher scores indicate a stronger SOC. In the present study, the Cronbach's α was 0.825.

#### Perceived partner responsiveness (PPRS)

Participants' feelings of being understood, validated, and cared for by their partners^20^ were measured using the 12-item PPRS. Items are rated on a seven-point Likert scale (1 = not at all true to 7 = completely true), with higher scores indicating greater perceived responsiveness. In the current study, the Cronbach's *α* was 0.976.

#### Distress disclosure (DD)

The Distress Disclosure Index (DDI) was employed to assess the tendency to disclose personally distressing information.^27^ It consists of 12 items rated on a 5-point Likert scale (1 = strongly disagree, 5 = strongly agree), with total scores ranging from 12 to 60, where higher scores indicate a greater willingness to disclose distress.^27^ In the current study, the Cronbach's *α* was 0.980.

#### Financial toxicity (COST)

Financial toxicity was assessed using the Comprehensive Score for Financial Toxicity (COST) instrument.^8^ This 11-item scale evaluates financial strain across three domains: expenses, resources, and psychosocial impact. Items are rated on a five-point Likert scale ranging from 0 (not at all) to 4 (very much). Items 1, 6, 7, and 11 are positively scored, while the remaining items are reverse-scored. The total score ranges from 0 to 44, with higher COST scores indicating lower financial toxicity.^8^ In this study, the Cronbach's *α* was 0.786.

#### Perceived social support Scale (PSSS)

The 12-item Perceived Social Support Scale (PSSS)^28^ was used to assess individuals' perceived social support from family, relatives, colleagues, and friends. The items are rated on a seven-point Likert scale, with higher scores indicating better perceived social support. Scores less than or equal to 36 indicate low perceived support, scores between 37 and 60 indicate moderate support, and scores between 61 and 84 indicate high support.^28^ In the present study, the scale showed high internal consistency, with a Cronbach's *α* of 0.901.

### Survey procedure

Following ethical approval, researchers coordinated with relevant hospital departments and trained research assistants to screen inpatients based on predefined inclusion and exclusion criteria. Eligible participants received standardized one-on-one study explanations, and written informed consent was obtained prior to participation.

Data were collected via an electronic questionnaire administered through the Wenjuanxing platform. Participants completed the survey independently by scanning a QR code on a smartphone or a researcher-provided tablet in a private setting. A prior pilot test with 15 patients indicated that a careful and attentive reading of all scale items required approximately 10–15 minutes. Research assistants were available for technical clarification but did not influence responses. All items were mandatory, and questionnaires were submitted anonymously and in real time to a secure, encrypted server.

Of the 300 patients approached, 34 were excluded based on rigorous data quality screening. Specifically, 23 cases were removed due to insufficient response time, which was operationally defined as a completion time of less than 300 seconds (5 minutes), a threshold established based on our pilot test, as completing the survey in less than half of the minimum required time indicated a lack of attentive cognitive processing.^29^ Additionally, 11 cases were identified as duplicate entries and excluded; these were detected by screening for identical and highly overlapping response vectors, wherein participants selected exactly the same options for all demographic, clinical, and psychometric scale items, indicating redundant or non-independent submissions, resulting in a final sample of 266 participants (effective response rate: 88.7%).

### Statistical analysis

Analyses were performed using SPSS 26.0 and RStudio. Continuous variables were presented as mean ± standard deviation (SD), non-normally distributed variables as median and interquartile range [M (Q1, Q3)], and categorical variables as frequencies and percentages (*n*, %).

Univariate analyses were then performed to identify factors associated with TP dimensions. Independent samples *t*-tests or one-way ANOVA were used for categorical variables, and Pearson correlation analysis was applied for continuous variables. Variables with *P* < 0.05 in univariate analyses were entered into subsequent multivariate analyses. Pearson correlation analysis was also conducted to examine the inter-correlations among the five TP dimensions prior to the main regression analyses.

Finally, five multivariate linear regression models were constructed to identify associated factors of each TP dimension. TP dimension scores were treated as dependent variables, and statistically significant variables from univariate analyses were included as independent variables using the Enter method. Prior to multivariable regression analyses, all multi-level categorical variables (residence, education level, occupation, employment status, per capita monthly household income, medical insurance type, cancer diagnosis, cancer stage, and treatment phase) were dummy-coded, with reference categories selected based on clinical relevance and sample distribution. All tests were two-tailed, with a significance level of *α* = 0.05. To control the False Discovery Rate (FDR) arising from multiple testing across the five multivariable regression models, the Benjamini-Hochberg procedure was applied to the *P*-values of all primary predictors and multi-categorical dummy variables. A corrected false discovery rate (*Q*-value < 0.05) was considered statistically significant for the robust identification of associated factors.

## Results

### Demographic characteristics

A total of 266 patients with gynecologic malignancies were enrolled, with a mean age of 43.80 ± 4.77 years. Cervical cancer was the most prevalent diagnosis (57.1%), and 66.5% of participants were undergoing active treatment. Regarding socioeconomic distribution, 39.1% and 26.3% of the participants resided in urban and rural areas, respectively. Nearly 40% had completed primary or junior secondary education, with the majority employed in manual (35.0%) or non-manual labor (29.7%). The predominant insurance type was rural/urban insurance (54.1%). Detailed characteristics are summarized in Table 1.

**Table 1:** Demographic and clinical characteristics of participants (*N* = 266)

Variables	Total, *n* (%)	Variables	Mean ± SD
Residence			
Urban city	104 (39.1)	Age (years)	43.80 ± 4.77
County town	70 (26.3)	Number of children	1.50 ± 0.61
Town	22 (8.3)	Age of the youngest child	12.23 ± 4.15
Rural village	70 (26.3)	Parenting concerns	2.42 ± 0.65
Education level		Sense of coherence	60.32 ± 10.77
Primary/Junior high	104 (39.1)	Distress disclosure index	43.71 ± 12.67
Senior high/Vocational	90 (33.8)	Perceived social support	66.42 ± 15.25
University or above	72 (27.1)	Perceived partner responsiveness	67.03 ± 13.87
Occupation		Financial toxicity	20.99 ± 5.97
Intellectual labor	79 (29.7)		
Manual labor	93 (35.0)		
Freelance	47 (17.7)		
Retired/Unemployed	47 (17.7)		
Employment status			
Employed	61 (22.9)		
Resigned	110 (41.4)		
Take leave	95 (35.7)		
Medical insurance			
Employee/Commercial	113 (42.5)		
Resident (Rural/Urban)	144 (54.1)		
Self-pay	9 (3.4)		
Family structure			
Nuclear family	214 (80.5)		
Non-nuclear	52 (19.5)		
Cancer diagnosis			
Cervical cancer	152 (57.1)		
Ovarian cancer	72 (27.1)		
Endometrial cancer	24 (9.0)		
Others	18 (6.8)		
Cancer stage			
Stage I	76 (28.6)		
Stage II	111 (41.7)		
Stage III	73 (27.4)		
Stage IV	6 (2.3)		
Treatment phase			
Active treatment	177 (66.5)		
Stable/Observation	80 (30.1)		
Palliative/Recurrence	9 (3.4)		
Per capita monthly household income (¥)			
Low (< 3000)	86 (32.3)		
Medium (3000–5000)	90 (33.8)		
High (> 5000)	90 (33.8)		

SD, standard deviation. Treatment phase reflects clinical management strategy, not disease duration.

### Status of time perspective (TP) dimensions

Descriptive statistics for the five TP dimensions are presented in Table 2. All dimensions exhibited variability and followed a normal distribution. The mean score for the Past-Negative dimension was 2.85 (SD = 0.63), while the Past-Positive dimension showed a mean score of 3.65 (SD = 0.53). For the Present-Hedonistic dimension, the mean score was 2.89 (SD = 0.78), and the Present–Fatalistic dimension had a mean score of 3.13 (SD = 0.79). The Future dimension demonstrated a mean score of 3.62 (SD = 0.65). Correlations between the five dimensions ranged from *r* = −0.600 to *r* = 0.319 (Table 3).

**Table 2:** Time perspective dimension scores among patients with gynecologic malignancies (*N* = 266)

Time perspective category	Mean	Standard deviation	Minimum value	Maximum value	Skewness	Kurtosis
Past-negative	2.85	0.63	1.29	4.00	−0.06	−0.70
Past-positive	3.65	0.53	2.17	4.67	−0.76	0.21
Present-hedonistic	2.89	0.78	1.25	4.25	−0.02	−1.21
Present–fatalistic	3.13	0.79	1.67	5.00	0.30	−0.45
Future	3.62	0.65	1.40	4.90	−0.89	0.34

**Table 3:** Pearson correlation matrix of the five time perspective dimensions (*N* = 266)

TP Dimension	Past Negative	Past Positive	Present Hedonistic	Present Fatalistic	Future
Past negative	–				
Past positive	0.026 *P* = 0.678	–			
Present hedonistic	0.314∗∗∗ *P* < 0.001	0.061 *P* = 0.323	–		
Present fatalistic	0.319∗∗∗ *P* < 0.001	0.011 *P* = 0.852	0.251∗∗∗ *P* < 0.001	–	
Future	−0.232∗∗∗ *P* < 0.001	−0.021 *P* = 0.731	−0.488∗∗∗ *P* < 0.001	−0.600∗∗∗ *P* < 0.001	–

∗*P* < 0.05; ∗∗*P* < 0.01; ∗∗∗*P* < 0.001 (two-tailed).

### Univariate and correlation analyses

Past-Negative: Scores varied significantly by residence, educational level, occupation, employment status, medical insurance, treatment phase, and per capita monthly household income. Furthermore, they were negatively correlated with SOC, DD, PSSS, PPPS and COST, but positively associated with PCQ.

Past-Positive: Significant differences were observed only regarding residence and cancer stage.

Present-Hedonistic: Scores were positively correlated with age, number of children, and age of the youngest child; and negatively correlated with SOC, DD and COST. Significant group differences emerged regarding residence, education level, occupation, employment status, medical insurance, cancer diagnosis, cancer stage, and per capita monthly household income.

Present–Fatalistic: Scores differed significantly across residence, education level, occupation, employment status, medical insurance, cancer stage and per capita monthly household income. Significant negative correlations were found with SOC, DD, PSSS, PPRS, and COST, and positive correlations with the number of children.

Future: This dimension was associated with residence, education level, occupation, employment status, medical insurance, cancer stage and per capita monthly household income. It was negatively correlated with the number of children and the age of the youngest child, but positively correlated with SOC, DD, PSSS, PPRS, and COST (Supplementary Table S1).

### Regression analysis of factors associated with TP

Prior to the multivariable linear regression analysis, key assumptions were evaluated to ensure model validity. Multicollinearity tests yielded Variance Inflation Factors (VIF) ranging from 1.083 to 2.899, confirming the absence of significant multicollinearity. Independence of residuals was verified via scatterplots of standardized residuals, which displayed a random distribution. Furthermore, normality and homogeneity of variance were confirmed through P–P plots and residual scatterplots, respectively; both assessments indicated that the data satisfied the fundamental requirements for linear regression analysis.

Patients residing in county towns or rural villages reported significantly higher Past-Negative scores relative to those living in urban cities. Compared with women who had a university education, those with primary/junior high school education and senior high school education reported significantly lower Past-Negative scores. Meanwhile, employed patients had lower Past-Negative scores, higher PSSS and PCQ scores were positively associated with Past-Negative orientation. Conversely, higher SOC, DD, and PPRS scores were significantly negatively associated with Past-Negative orientation.

Compared with the urban reference group, living in rural villages was associated with significantly lower Past-Positive scores. In terms of clinical features, patients diagnosed with Stage II and Stage III malignancies demonstrated significantly higher Past-Positive orientation than those at Stage I.

Patients residing in towns or rural villages reported significantly higher Present-Hedonistic scores than those living in urban cities. Lower educational attainment (primary/junior high) was negatively associated with this dimension. Regarding occupation, manual labor and freelance work were positively associated with scores compared with intellectual labor. Conversely, being resigned, having resident medical insurance, and having ovarian cancer were negatively associated with this dimension. Among continuous variables, a greater number of children and higher DD scores were positively associated with Present-Hedonistic orientation, whereas higher SOC scores were negatively associated with it.

Relative to urban city dwellers, patients living in county towns scored lower on the Present-Fatalistic scale. Compared to intellectual laborers, individuals engaged in manual labor exhibited significantly higher scores, as did those in the retired or unemployed cohorts. Regarding psychological and systemic correlates, an increased SOC, higher PSSS, and higher scores in PPRS and COST were strongly linked to lower fatalistic tendencies. Additionally, higher DD scores were positively associated with this profile.

Residents of towns and rural villages exhibited significantly lower Future orientation scores compared to those in urban cities, while patients living in county towns exhibited higher Future orientation. Manual labor and retired/unemployed status were associated significantly with lower Future orientation scores relative to intellectual laborers. In terms of clinical features, patients diagnosed with Stage II malignancies demonstrated significantly lower Future orientation than those at Stage I. For psychological and clinical characteristics, higher SOC and PSSS were positively associated with stronger future orientation (Table 4).

**Table 4:** Multivariable linear regression models for five time perspective dimensions (*N* = 266)

Variable	Past-Negative		Past-Positive		Present-Hedonistic		Present–Fatalistic		Future	
	B (SE)	*P* value	B (SE)	*P* value	B (SE)	*P* value	B (SE)	*P* value	B (SE)	*P* value
Residence (ref: Urban city)										
County town	0.307 (0.100)	0.002∗∗	−0.092 (0.083)	0.269	0.226 (0.127)	0.076	−0.239 (0.119)	0.046∗	0.275 (0.090)	0.002∗∗
Town	0.264 (0.148)	0.075	0.228 (0.121)	0.062	0.497 (0.185)	0.008∗∗	0.004 (0.177)	0.983	−0.311 (0.134)	0.021∗
Rural village	0.408 (0.144)	0.005∗∗	−0.213 (0.083)	0.011∗	0.788 (0.181)	< 0.001∗∗∗	−0.109 (0.170)	0.521	−0.279 (0.129)	0.031∗
Education level (ref: University)										
Primary/Junior	−0.292 (0.130)	0.025∗	–	–	−0.384 (0.171)	0.025∗	−0.040 (0.152)	0.791	−0.099 (0.116)	0.395
Senior high	−0.199 (0.101)	0.049∗	–	–	−0.176 (0.131)	0.180	−0.201 (0.117)	0.097	−0.039 (0.091)	0.670
Occupation (ref: Intellectual labor)										
Manual labor	0.151 (0.112)	0.178	–	–	0.410 (0.137)	0.003∗∗	0.513 (0.131)	< 0.001∗∗∗	−0.483 (0.099)	< 0.001∗∗∗
Freelance	−0.230 (0.131)	0.081	–	–	0.402 (0.169)	0.018∗	0.306 (0.158)	0.053	−0.051 (0.119)	0.668
Retired/Unemployed	0.128 (0.150)	0.394	–	–	0.307 (0.190)	0.107	0.740 (0.178)	< 0.001∗∗∗	−0.288 (0.134)	0.033∗
Employment status (ref: Take leave)										
Employed	−0.224 (0.101)	0.027∗	–	–	−0.239 (0.144)	0.099	−0.177 (0.119)	0.137	−0.024 (0.103)	0.815
Resigned	−0.013 (0.120)	0.916	–	–	−0.573 (0.160)	< 0.001∗∗∗	−0.265 (0.136)	0.053	0.052 (0.113)	0.643
Per capita monthly household income (ref: High)										
Low	−0.267 (0.137)	0.053	–	–	−0.069 (0.171)	0.688	0.166 (0.160)	0.302	0.082 (0.120)	0.496
Medium	−0.098 (0.098)	0.318	–	–	−0.137 (0.122)	0.264	0.211 (0.115)	0.068	0.009 (0.086)	0.914
Medical insurance (ref: Employee)										
Resident	−0.103 (0.120)	0.393	–	–	−0.359 (0.147)	0.019∗∗	−0.014 (0.143)	0.923	−0.002 (0.106)	0.983
Self-pay	0.242 (0.217)	0.265	–	–	−0.370 (0.267)	0.166	−0.115 (0.251)	0.647	0.132 (0.190)	0.486
Cancer stage (ref: Stage I)										
Stage II	–	–	0.275 (0.077)	< 0.001∗∗∗	0.1142 (0.100)	0.157	0.003 (0.094)	0.977	−0.219 (0.071)	0.002∗∗
Stage III	–	–	0.180 (0.088)	0.043∗	−0.230 (0.128)	0.073	−0.178 (0.115)	0.125	−0.120 (0.087)	0.171
Stage IV	–	–	0.238 (0.223)	0.286	−0.592 (0.310)	0.058	0.072 (0.282)	0.798	0.154 (0.220)	0.478
Treatment phase (ref: Active)										
Stable	−0.117 (0.077)	0.127	–	–	–	–	–	–	–	–
Palliative	−0.097 (0.182)	0.595	–	–	–	–	–	–	–	–
Cancer diagnosis										
Ovarian	–	–	–	–	0.248 (0.100)	0.014∗				
Endometrial	–	–	–	–	0.244 (0.154)	0.114	–	–		
Others	–	–	–	–	0.004 (0.191)	0.984	–	–	–	–
Continuous variables										
No. of children					0.163 (0.079)	0.041∗	0.031 (0.073)	0.673	0.022 (0.055)	0.685
Age of the youngest child					0.007 (0.012)	0.590	–	–	0.003 (0.008)	0.737
Age					0.010 (0.011)	0.352				
SOC	−0.018 (0.004)	< 0.001∗∗∗	–	–	−0.020 (0.005)	< 0.001∗∗∗	−0.017 (0.005)	< 0.001∗∗∗	0.020 (0.004)	< 0.001∗∗∗
DD	−0.012 (0.004)	0.004∗∗	–	–	0.009 (0.004)	0.043∗	0.014 (0.005)	0.007∗∗	0.000 (0.004)	0.949
PSSS	0.014 (0.004)	< 0.001∗∗∗	–	–	–	–	−0.019 (0.004)	< 0.001∗∗∗	0.006 (0.003)	0.039∗
PPRS	−0.008 (0.003)	0.013∗	–	–	–	–	−0.008 (0.004)	0.025∗	–	–
PCQ	0.201 (0.062)	0.001∗∗	–	–	–	–	–	–	–	–
COST	−0.008 (0.003)	0.253	–	–	−0.001 (0.009)	0.917	−0.019 (0.008)	0.016∗	−0.012 (0.006)	0.056
R^2^	0.407		0.085		0.424		0.486		0.561	
Adjusted R^2^	0.353		0.064		0.361		0.437		0.520	
F (*P*)	7.580 (< 0.001)		4.027 (0.001)		6.762 (< 0.001)		9.928 (< 0.001)		13.460 (< 0.001)	

∗*P* < 0.05; ∗∗*P* < 0.01; ∗∗∗*P* < 0.001 (two-tailed). “-" indicates that the variable was not included in the model due to a non-significant result in the univariate analysis.

SOC, Sense of Coherence; DD, Distress Disclosure; PSSS, Perceived Social Support Scale; PPRS, Perceived partner responsiveness; PCQ, Parenting Concern Questionnaire; COST, Comprehensive Score for Financial Toxicity.

All *P*-values are unadjusted (raw) *P*-values. To control the False Discovery Rate (FDR) arising from multiple testing, the Benjamini-Hochberg procedure was applied across all models; all primary significant findings remained robust with a corrected false discovery rate (*Q*-value) of < 0.05.

## Discussion

### Main findings

This study investigated the TP dimensions among a specific cohort of Chinese patients with gynecologic malignancies who have partnering and parenting responsibilities. To our knowledge, this is the first study to delineate variations in TP and its associated factors within this specific population, providing an integrative perspective for understanding their complex psychological experiences.

Our study indicates that young and middle-aged patients with gynecologic malignancies exhibited distinct TP dimensions. Specifically, scores were higher for the Past-Positive and Future dimensions (3.65 ± 0.53 and 3.62 ± 0.65), while Past-Negative and Present-Hedonistic scores were lower (2.85 ± 0.63 and 2.89 ± 0.78), and Present–Fatalistic scores were at a moderate level (3.13 ± 0.79). A strong Past-Positive orientation, characterized by reconstructing life through a meaningful lens, may facilitate psychological adaptation by helping patients reframe their illness narratives.^12^ Conversely, the subdued Past-Negative orientation indicates reduced tendency to dwell on adverse events, thereby contributing to more effective emotional regulation.^13^ These results are consistent with existing research; for instance, Moskalewicz et al.^9^ observed similar patterns in general cancer populations, with elevated Past-Positive and Future scores alongside lower Past-Negative and Present-Hedonistic scores. Both TP theory^14^ and empirical studies^13^^,^^30^ indicate that high Past-Positive and Future scores correlate with greater psychological well-being and health-promoting behaviors. The TP status observed in our sample of gynecologic malignancy patients thus aligns with broader cancer populations, suggesting that this group retains important cognitive resources conferring psychological resilience when facing adversity. Although the five TP dimensions were analyzed separately to facilitate interpretation, we acknowledge their theoretical inter-relatedness as documented in prior literature. Future studies may consider multivariate approaches such as MANOVA to simultaneously model all dimensions and account for their covariance structure.

In the Past-Negative dimension, higher PSSS was associated with higher Past-Negative scores, contrary to theoretical predictions.^17^^,^^31^ One possible explanation is that patients experiencing stronger negative appraisals of past illness experiences may receive or perceive greater support from family members and social networks. Given the cross-sectional nature of this study, the directionality of this association remains unclear and warrants further investigation. Lower SOC is linked to difficulties in the cognitive integration of the cancer diagnosis, which closely correlates with patients being entangled in aversively perceived past experiences.^19^^,^^26^ The link between lower distress disclosure and higher Past-Negative scores may reflect an emotion-suppressing coping style prevalent in Chinese cultural contexts,^32^ as individuals from Asian backgrounds often prioritize emotional suppression to maintain interpersonal harmony.^33^ Notably, lower educational attainment was linked to lower Past-Negative focus; a possible explanation is that women with higher educational levels may possess greater disease awareness and engage in more extensive cognitive appraisal of illness-related losses and disruptions. Such reflective processing may increase the salience of negative past experiences, thereby strengthening Past-Negative orientation.^34^ Furthermore, actively employed patients reported lower Past-Negative scores than those on leave, suggesting that maintaining occupational continuity preserves pre-illness social roles and distracts patients from ruminating on negative historical appraisals.^35^ The significant association between poorer partner responsiveness and higher Past-Negative focus implies that a lack of dyadic validation is closely linked to a heightened perception of historical trauma. Higher parenting concerns were associated with stronger Past-Negative focus, as chronic worries about children's futures may trigger painful recollections of illness consequences.^36^ Non-urban residence may reflect structural disadvantages such as limited health care access that could intensify negative past appraisals.^37^^,^^38^ Additionally, lower COST scores (indicating higher financial toxicity) may be associated with greater rumination on the financial damages already incurred since diagnosis.^8^

Rural village residence was negatively associated with Past-Positive scores. This finding may reflect the cumulative burden of structural disadvantages in rural settings, such as limited health care access and economic strain,^39^ which may overshadow the potentially protective effects of place identity.^40^ Future research should explore whether rural-urban disparities in resource availability differentially shape temporal orientation patterns. Stage II and III patients reported higher Past-Positive scores than Stage I patients, potentially reflecting that individuals facing a greater disease burden may be more likely to draw upon positive memories as adaptive coping resources.^9^ Such a pattern is broadly consistent with theories of positive cognitive reappraisal, whereby individuals may reconstruct meaning and derive psychological strength from prior life experiences when confronted with significant health challenges.^41^

Regarding the Present-Hedonistic dimension, county, town and rural village residence also showed positive associations compared with urban settings. This stronger present focus may stem from non-urban life uncertainties, such as limited health care access, less employment stability, and fewer future opportunities, which may lead individuals to prioritize immediate well-being over distant goals.^42^ Having more children may increase hedonic focus, as the daily demands of parenting anchor patients firmly in the immediate present, leaving little cognitive bandwidth for distal temporal horizons.^43^ Moreover, manual labor, freelance work, and retired or unemployed status were associated with higher Present-Hedonistic scores. These findings suggest that less stable occupational circumstances and changes in social roles may promote a stronger focus on immediate experiences rather than long-term planning.^44^ According to Danigno's review,^45^ patients with lower SOC may abandon long-term planning and instead seek immediate gratification as a coping mechanism. The positive link between higher distress disclosure and hedonism suggests that emotionally expressive patients may rely on present-oriented verbal release to cope with current suffering.^27^

As for the Present–Fatalistic dimension, the simultaneous deficits in internal resources (SOC) and external support resources (PSSS, PPRS) were associated with a fatalistic worldview, consistent with learned helplessness theory.^46^ Furthermore, freelance status^47^ and manual labor may be intertwined with fatalistic thinking, which may stem from a profound sense of vulnerability following the loss of active social contributions and physical control over one's daily routine.^48^ Lower COST scores (indicating higher financial toxicity) were strongly associated with higher fatalism, as economic hardship may reduce perceived autonomy and control, two key psychological resources underlying adaptive coping. When financial demands are viewed as overwhelming and difficult to manage, patients may increasingly perceive life circumstances as externally determined, which is closely linked to a more fatalistic temporal outlook.^49^^,^^50^ Unexpectedly, higher distress disclosure was associated with higher fatalism; this counterintuitive finding may reflect that high-distress individuals are simultaneously prone to both disclosure and fatalistic thinking,^51^ though the directionality of this relationship warrants further investigation.

Higher SOC and stronger PSSS were positively associated with future-oriented thinking, which may be linked to a higher information-processing capacity and a stronger belief that the future is comprehensible and manageable.^31^^,^^52^^,^^53^ Compared with urban residents, patients living in county towns demonstrated higher Future orientation, whereas those residing in towns and rural villages exhibited lower Future scores. This pattern may suggest that county-level settings provide a balance between health care accessibility and social connectedness, which may support future-oriented thinking.^39^ In contrast, residents of towns and rural villages may face greater resource constraints and treatment-related uncertainties that hinder long-term planning and future-oriented perspectives.^42^^,^^54^ Conversely, manual labor and retirement or unemployment were associated with lower Future scores, suggesting that occupational disruption may be linked to reduced long-term planning capacity. The mechanisms underlying these associations warrant further investigation in future research.

Notably, while the Benjamini-Hochberg FDR correction was utilized to mitigate the risk of false positives, the complex nature of multivariable regressions with numerous psychosocial variables means that the risk of Type I errors cannot be entirely eliminated. Therefore, the observed associated factors should be interpreted as tentative indicators rather than definitive determinants. Collectively, these findings help explain the heterogeneity in psychological responses observed among patients with similar clinical profiles. The distinct profiles identified across TP dimensions suggest that temporal orientation may play a role in driving individual differences, highlighting the potential value of TP-informed interventions in this population.^13^^,^^52^

### Implications for nursing practice and research

Our findings offer critical insights for psychosocial interventions in young and middle-aged gynecologic cancer care. First, rather than merely treating negative symptoms, clinicians should prioritize enhancing SOC through meaning-centered therapy and cognitive restructuring to mitigate Past-Negative bias. For those with high parenting concerns, future-oriented goal-setting can effectively reframe roles and improve temporal adaptation. Second, the buffering effect of partner responsiveness against Present-Fatalism underscores the necessity of dyadic interventions, such as communication training and structured disclosure sessions, to foster mutual validation. Finally, we identified specific risk clusters for targeted screening, enabling a shift toward stratified, precision psychosocial support. Adopting a socio-ecological approach, interventions should be implemented across individual (cognitive strategies), interpersonal (partner support), and organizational (routine screening) levels to address the multi-level associated factors of temporal adaptation.

### Limitations

This study has several limitations. First, its cross-sectional design precludes the establishment of causal relationships among variables. Second, the convenience sample from three provincial hospitals in a single region, which may limit the generalizability of our findings. This selective cohort focused on married women with parenting responsibilities, meaning findings may not apply to unmarried, childless, or outpatient subgroups, and observed urban-rural differences require validation in more diverse cohorts. Third, using univariate screening for variable selection might have excluded potential confounders, affecting regression stability. Fourth, certain clinical and socioeconomic subgroups had small cell sizes, such as Stage IV (*n* = 6) and self-pay (*n* = 9), which compromises statistical power, so these findings must be interpreted with caution. Furthermore, oncology treatments impose a rigid, cyclical structure on patients that may disrupt their natural temporal orientation, and the ZTPI does not account for this circular temporality.^55^ Finally, some influential variables, such as personality traits and specific coping strategies, were not included, necessitating more comprehensive models in future research.

## Conclusions

In conclusion, this study reveals that young and middle-aged women with gynecologic malignancies who are married and raising minor children exhibit a distinct and imbalanced time perspective, characterized by a stronger focus on a positive past and a weaker orientation towards a negative past or present hedonism. Our findings underscore the critical role of both internal psychological resources, such as a strong SOC, and external relational resources, like robust social and partner support, as important correlates of a more adaptive and future-oriented temporal framework. These results highlight the clinical utility of time perspective as a lens for understanding psychological adaptation and suggest that psychosocial interventions aimed at strengthening SOC and enhancing support systems could be a promising avenue for promoting resilience in this vulnerable patient population. The integration of multivariable findings revealed a distinct temporal profile among young and middle-aged women with gynecologic cancer. Past-Negative and Present–Fatalistic orientations were characterized by diminished psychological and social resources, while Future orientation reflected a more adaptive perspective supported by stronger SOC and social support. Financial toxicity and distress disclosure were particularly relevant to Present–Fatalistic orientation. In contrast, Past-Positive orientation was mainly influenced by residential and clinical characteristics, whereas Present-Hedonistic orientation appeared to reflect a present-focused response associated with socioeconomic and psychosocial challenges.

## CRediT authorship contribution statement

**Guiyuan Ma:** Conceptualization, Methodology, Performed the data analysis, Writing-Original draft. **Cai Deng & Qiming Ding:** Writing - review & editing, Data collection and Supervision. **Can Gu:** Software, Validation. **Juan Peng:** Project administration, Funding acquisition. All authors have read and approved the final manuscript.

## Ethics statement

Approval was obtained from the Institutional Review Board of Xiangya School of Nursing, Central South University (Approval No. E202430). The procedures used in this study adhere to the tenets of the Declaration of Helsinki. All participants provided written informed consent.

## Data availability statement

The datasets generated during and/or analyzed during the current study are available from the corresponding author upon reasonable request.

## Declaration of generative AI and AI-assisted technologies in the writing process

No AI tools/services were used during the preparation of this work.

## Funding

This work was supported by National Natural Science Foundation of China (82272924) and China Community Health Services Program (2023YC01). The funders had no role in considering the study design or in the collection, analysis, interpretation of data, writing of the report, or decision to submit the article for publication.

## Declaration of competing interest

The authors declare no conflict of interest. The fourth author, Prof. Can Gu, is an editorial board member of *Asia–Pacific Journal of Oncology Nursing*. The article was subject to the journal's standard procedures, with peer review handled independently of Prof. Gu and her research groups.
