# Supplementary material for: Time perspective status and associated factors among young and middle-aged women with gynecologic malignancies who have parenting responsibilities
Source: Asia Pac J Oncol Nurs. 2026 Jun 13;13:100994. doi: 10.1016/j.apjon.2026.100994 (PMC13319719; doi:10.1016/j.apjon.2026.100994)
Supplement: Multimedia component 1 [file mmc1.docx]

**Table S1** Univariate and Correlation Analyses of Time Perspective Dimensions and Related Variables of the Participants

| **Variables** | **Past-Negative** | |  | **Past-Positive** | |  | **Present-Hedonistic** | |  | **Present-Fatalistic** | |  | **Future** | |
| --- | --- | --- | --- | --- | --- | --- | --- | --- | --- | --- | --- | --- | --- | --- |
|  | **Test statistic** | ***P* value** |  | **Test statistic** | ***P* value** |  | **Test statistic** | ***P* value** |  | **Test statistic** | ***P* value** |  | **Test statistic** | ***P* value** |
| Residence ^b^ | 29.955 | <0.001^***^ |  | 10.797 | 0.013^*^ |  | 59.408 | <0.001^***^ |  | 32.495 | <0.001^***^ |  | 78.559 | <0.001^***^ |
| Education Level ^b^ | 7.329 | 0.026^*^ |  | 2.802 | 0.063 |  | 31.651 | <0.001^***^ |  | 35.662 | <0.001^***^ |  | 53.102 | <0.001^***^ |
| Occupation ^b^ | 44.525 | <0.001^***^ |  | 1.903 | 0.129 |  | 44.517 | <0.001^***^ |  | 65.561 | <0.001^***^ |  | 73.718 | <0.001^***^ |
| Employment status ^b^ | 11.564 | 0.003^**^ |  | 2.520 | 0.284 |  | 37.225 | <0.001^***^ |  | 29.550 | <0.001^***^ |  | 31.556 | <0.001^***^ |
| Medical Insurance ^b^ | 10.533 | 0.005^**^ |  | 1.730 | 0.421 |  | 13.104 | <0.001^***^ |  | 38.703 | <0.001^***^ |  | 27.584 | <0.001^***^ |
| Family Structure ^a^ | -1.212 | 0.226 |  | -0.913 | 0.362 |  | 0.086 | 0.931 |  | -0.094 | 0.925 |  | -1.288 | 0.199 |
| Cancer Diagnosis ^b^ | 2.138 | 0.544 |  | 0.746 | 0.862 |  | 12.950 | 0.005^**^ |  | 6.555 | 0.088 |  | 7.040 | 0.071 |
| Cancer Stage ^b^ | 4.074 | 0.254 |  | 11.232 | 0.011^*^ |  | 9.021 | 0.029^*^ |  | 9.697 | 0.021* |  | 19.827 | <0.001^***^ |
| Treatment Phase ^b^ | 8.933 | 0.011^*^ |  | 0.408 | 0.816 |  | 5.147 | 0.076 |  | 0.680 | 0.712 |  | 4.06 | 0.131 |
| Per Capita Monthly Household Income (CNY) ^b^ | 21.672 | <0.001^***^ |  | 2.592 | 0.274 |  | 41.078 | <0.001^***^ |  | 65.858 | <0.001^***^ |  | 52.010 | <0.001^***^ |
| Age ^c^ | -0.047 | 0.448 |  | -0.006 | 0.926 |  | 0.266 | <0.001^***^ |  | 0.033 | 0.630 |  | -0.114 | 0.064 |
| Number of Children ^c^ | 0.069 | 0.260 |  | 0.060 | 0.330 |  | 0.301 | <0.001^***^ |  | 0.170 | 0.005^**^ |  | -0.264 | <0.001^***^ |
| Age of the Youngest Child ^c^ | 0.091 | 0.138 |  | -0.020 | 0.747 |  | 0.231 | <0.001^***^ |  | 0.030 | 0.630 |  | -0.178 | 0.004^**^ |
| Parenting concern ^c^ | 0.293 | <0.001^***^ |  | 0.078 | 0.207 |  | -0.025 | 0.680 |  | 0.053 | 0.388 |  | -0.069 | 0.260 |
| Sense of Coherence ^c^ | -0.394 | <0.001^***^ |  | -0.016 | 0.795 |  | -0.293 | 0.001^***^ |  | -0.487 | <0.001^***^ |  | 0.496 | <0.001^***^ |
| Distress Disclosure Index ^c^ | -0.327 | <0.001^***^ |  | -0.058 | 0.345 |  | -0.145 | 0.018^*^ |  | -0.355 | <0.001^***^ |  | 0.387 | <0.001^***^ |
| Perceived social support ^c^ | -0.158 | 0.010^*^ |  | 0.109 | 0.076 |  | -0.080 | 0.195 |  | -0.503 | <0.001^***^ |  | 0.444 | <0.001^***^ |
| Perceived partner responsiveness ^c^ | -0.259 | <0.001^***^ |  | 0.086 | 0.160 |  | -0.035 | 0.573 |  | -0.459 | <0.001^***^ |  | 0.390 | <0.001^***^ |
| Financial Toxicity ^c^ | -0.355 | <0.001^***^ |  | 0.030 | 0.622 |  | -0.121 | 0.049^*^ |  | -0.384 | <0.001^***^ |  | 0.194 | 0.001^**^ |
| Note: a: t-test; b: Kruskal-Wallis H test; c: Correlation coefficient r; ^*^*P*<0.05; ^**^*P*<0.01; ****P*<0.001 (two-tailed). | | | | | | | | | | | | | | |
